# Obstetricians and midwives perspective of the alarming high cesarean section rates in Greece and worldwide

**DOI:** 10.1016/j.heliyon.2024.e39177

**Published:** 2024-10-10

**Authors:** Lioumpov Tonakanian, Stamatios Petousis, Panagiotis Volteas, Aikaterini Karavida, Konstantinos Dinas, Theodoros Theodoridis, Alexandros Sotiriadis, Apostolos Athanasiadis

**Affiliations:** a Third Department of Obstetrics and Gynecology, Faculty of Medicine, Aristotle University of Thessaloniki, Thessaloniki, Greece; b Second Department of Obstetrics and Gynecology, Faculty of Medicine, Aristotle University of Thessaloniki, Thessaloniki, Greece; c First Department of Obstetrics and Gynecology, Faculty of Medicine, Aristotle University of Thessaloniki, Thessaloniki, Greece; d Department of Surgery, Renaissance School of Medicine, HSC T-12, Room 064, Stony Brook, NY, 11794, USA

**Keywords:** Cesarean, Delivery, Women's health issues, Midwifery, Education

## Abstract

**Introduction:**

This study aimed to outline the perspectives of obstetricians/gynecologists (physicians) and midwives regarding the alarmingly high rates of cesarean sections (CSs) to identify areas for improvement and describe the role of a regional obstetric quality initiative.

**Material and methods:**

A cross-sectional study was performed utilizing real-world data from questionaries provided to Greek midwives and obstetricians. Primary outcomes included the attitudes of Greek physicians and midwives toward the CS rates in Greece and around the world, as well as identifying potential solutions for lowering these rates. The secondary outcome was the potential correlation between the answers of the participants and their demographic parameters.

**Results:**

A total of 456 physicians and 234 midwives participated in the survey. Greek CS rates (>50 %) were considered “acceptable” and inevitable by 29.7 % and 32 % of the participants, respectively. Stratified analysis based on profession showed that significantly fewer midwives compared to obstetricians would agree with current CS rates. Participants who had obtained their degrees abroad were more likely to consider all CS rates more justified than physicians and midwives who had graduated from Greek medical schools. For all questions, the younger age subgroups responded in a way toward the non-acceptability of CS rates and favored the implementation of rules and practice control according to guidelines. The responses did not differ significantly between male and female physicians.

**Conclusions:**

Greek midwives, to a greater degree than obstetricians/gynecologists, consider the current CS rates unjustified and agree on the importance of implementing appropriate interventions to reduce them.

## Highlights/Statement of significance

• **Problem:** Despite the alarmingly high CS rates, the awareness of CS overutilization among healthcare professionals is limited and understudied.
• **What is Known:** Although there have been efforts to reduce the existing CS rates, the results highlight the complexity of the problem. dy to examine national variation in Greek health specialists' opinions of the alarming high CS rates.
• **What this Paper Add:** Greek midwives, to a greater degree than physicians, consider the current CS rates unjustified and agree on the importance of implementing appropriate interventions to reduce them.

## 1 Introduction

Cesarean section (CS) is considered a safe procedure, although compared to vaginal delivery, it has been associated with a 2- or 3 times higher risk of severe complications, including but not limited to bowel obstruction and incisional hernia, often requiring surgical repair [1]. Nonetheless, the rate of CSs for delivery has increased worldwide over the last 20 years and is now far higher than the optimal rate set by the World Health Organization (WHO) in 1985 (10–15 %) [2,3]. Specifically, the CS rate worldwide increased from 12 % in 2000 to 21 % in 2015 [2], while the CS rate across most European Union countries was greater than 15 % of all births in 2017 [4,5]. Although nationwide data are scarce in Greece**,** the reported CS rate in 2014 was over 53 % and 58 % in public and private hospitals, respectively. The CS remained dramatically high, peaking at 77 % in 2015 and back to 58 % in 2016 [6], placing Greece within the spectrum of countries with the highest CS rates worldwide [7,8].

It is well shown that overuse of CS is increasing around the world with harmful consequences to both individuals and healthcare systems, especially in low- and middle-income countries [9]. Hereby, the WHO has indicated that every effort should be made to provide CS to women in need rather than striving to achieve a specific rate, indicating that in places with rates below 9 %, socioeconomic development, rather than the CS rate, is the primary drive for morbidity and mortality rates. CS rates above 16 % do not further reduce maternal or perinatal mortality [10], and rates above 20 % outweigh the potential benefits that CS could offer [3,11,12].

However, country- and disease-specific data are limited, challenging any pragmatic efforts to allocate the utilization of resources from unnecessary interventions to areas of the health-care systems that need improvement (e.g., high-risk populations, underdiagnosed clinical entities). The high C-section rates in Greece reflect a global trend seen in many countries across the globe**.** Hence, comparison of practices between countries with similar socioeconomic status could provide valuable insight into strategies aiming to reduce CS rates [13]**.** This study aimed to record opinions and perspectives regarding CS rates among Greek healthcare professionals, including obstetricians/gynecologists as well as midwives, to investigate the awareness of high CS utilization. Additionally, an effort was made to compare these data to European standards and CS rates from other low- and middle-income countries around the globe and propose potential solutions.

## 2 Material and Methods

### 2.1 Study design and population

This cross-sectional study recorded the perspectives of obstetricians and midwives regarding CS rates in Greece and worldwide, as well as opinions about potential solutions for improving the CS rate. The survey was conducted using an anonymous online questionnaire (i.e., Google Form platform), and the target population was obstetricians/gynecologists and midwives working in Greece in public and private hospitals between April 2017 and July 2018. All participants had to have an active occupational license and be engaged in a related work arrangement with a specific employer.

An anonymous web-based survey was circulated to this group via email. If there was no response to the first invitation for participation in the survey, a second reminder was sent. All responses were de-identified, and only cumulative results or anonymous individual responses were presented. The survey was conducted in English and was closed for analysis in July 2018. To reduce participant bias, every subject could perform the survey only once, and the answers were locked upon submission.

### 2.2 Questionnaire development and completion

A broad questionnaire was developed based on pertinent questions created by a systematic review of relevant literature. The questions were then tailored to be more simple, targeted, and specific to the study's goal with the help of an expert committee. The experts' committee comprised five obstetricians/gynecologists with more than 20 years of clinical experience and strong knowledge of regional epidemiologic patterns of CSs and social determinants of health. All decisions were reached through extensive discussions and consensus with the rest of the research team. Then, the content validity ratio and content validity indices were calculated to assess the validity of each question quantitatively. Based on these results, the best set of questions was chosen. Table 1, Table 2, summarizes the final questions that were included in the survey.

**Table 1:** Question posed regarding perception and attitudes regarding Caesarean section.

PYLON I: PERCEPTIONS REGARDING INTERNATIONAL CS RATES	
**Q1**	Do you think CS rates in Greece (public hospitals 53.8 % and private clinics 58.7 % 2014) may be justified according to obstetrical science guidelines?
**Q2**	Do you think CS rates in European Union (average 25.3 %) may be justified according to obstetrical science guidelines?
**Q3**	Do you think CS rates in USA (2007–2014 average 33 %) may be justified according to obstetrical science guidelines?
**Q4**	Do you think CS rates in China (2007–2014 average 27 %) may be justified according to obstetrical science guidelines?
**Q5**	Do you think CS rates in Brazil (in 2014 average 57 % and private clinics 80–90 % in2011–2012) may be justified according to obstetrical science guidelines?
**Q6**	Do you think CS rates in Turkey (2007–2014 average 37 % and private clinics 66.7 % in 2014) may be justified according to obstetrical science guidelines?
**PERCEPTIONS REGARDING CS RATE IN GREECE**	
**Q7**	Do you think that the current CS rate in Greece is acceptable?
**Q8**	Do you think that the current CS rate in Greece is inevitable?
**Q9**	Should an effort be made to reduce CS rate in Greece?

**PYLON II:** **PERCEPTIONS REGARDING ACTIONS THAT MAY IMPROVE CS RATE IN GREECE** **(Do you think that mentioned actions may result in improvement of CS rates?)**	

**Q10**	Adopt minimum training credits to permit practicing obstetrics
**Q11**	Adopt regular examinations to continue practicing obstetrics
**Q12**	Adopt protocols and guidelines from relevant Colleges-Medical Companies (e.g., Hellenic Obstetrical and Gynecological Society)
**Q13**	Adopt protocols and guidelines from the government/state with legislation (e.g., Low Restricting Caesarean Births in Turkey, July 17, 2012)
**Q14**	Keeping guidelines under scrutiny by the government
**Q15**	Auditing personal records of obstetrical practice by authorized Medical Societies (Hellenic Obstetrical and Gynecological Society)
**Q16**	Perform regularly Vaginal Birth after Caesarean Section?
**Q17**	Rigorous control by Medical Associations on first CS indication current clinical practice

**Table 2:** Rates of positive answers on Questions 1–17 regarding perceptions and attitudes of both Physicians and midwives.

	Question	Positive answer (yes), n (%)
**Q1**	Greece CS rates justified	206 (29.7)
**Q2**	EU CS rates justified	466 (78.8)
**Q3**	USA CS rates justified	401 (69.6)
**Q4**	China CS rates justified	439 (78.1)
**Q5**	Brazil CS rates justified	131 (18.1)
**Q6**	Turkey CS rates justified	176 (25.4)
**Q7**	Greece CS rate acceptable	144 (20.7)
**Q8**	Greece CS rate inevitable	222 (32.0)
**Q9**	Effort to reduce Greece CS rate	545 (78.5)
**Q10**	Training credits to permit practicing	270 (38.9)
**Q11**	Regular examinations to continue practicing	425 (61.2)
**Q12**	Protocols & guidelines from colleges	608 (87.6)
**Q13**	Protocols & guidelines from government	454(65.4)
**Q14**	Keeping guidelines under scrutiny	365 (52.6)
**Q15**	Personal records of obstetrical practice by authorized Medical Societies	491 (70.7)
**Q16**	Perform VBAC	208 (30.0)
**Q17**	Control on first CS indication	454 (65.4)

### 2.3 Primary and secondary outcomes

The study's primary outcome was to assess the rates of positive responses regarding CS rates in Greece and worldwide and opinions toward potential solutions for reducing the CS rate. The secondary outcome was to investigate the possible correlation of answers with demographic aspects, including age, sex, education level, additional experience abroad, and having earned a Master of Science (MSc) and Ph.D. degree. Physicians were further requested to provide information regarding the country of specialty (Greece or abroad), the performance of any subspecialty, and the current performance of active obstetrical action.

### 2.4 Statistical analysis

Descriptive statistics were used for baseline demographic characteristics. Categorical variables are presented as absolute and relative frequencies (i.e., percentages) and were compared with the chi-square test (or Fisher's exact test when applicable). Numeric data are presented as mean ± standard deviation (SD) and compared using ANOVA or a paired *t*-test. In contrast, discrete data were compared with the Kruskal-Wallis test, or Wilcoxon signed rank test for paired data when appropriate. Linear regression analysis was also used to correlate the participants' answers with various epidemiological parameters of interest. The level of statistical significance was defined as a *P*-value <0.05. All statistical analyses were performed using SPSS version 15 (SPSS Inc., Chicago, IL, USA).

## 3 Results

### 3.1 Subject enrollment and baseline demographics

**Fig. 1:**
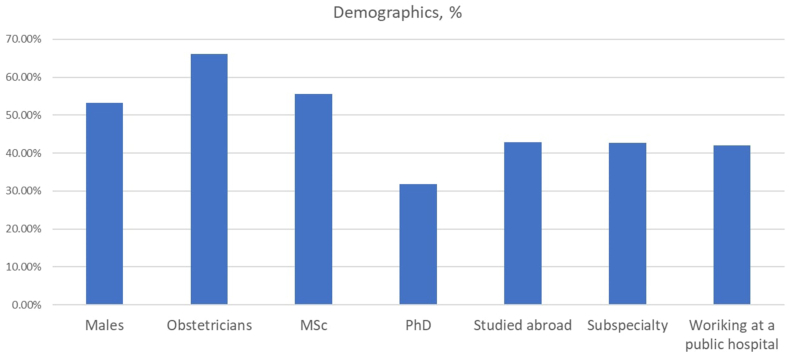
Baseline demographics of survey participants.

Overall, 690 subjects completed the assigned questionnaire, with slightly more than half of the participants being male practitioners (n = 367; 53.2 %). About 2 thirds of the enrolled subjects were obstetricians/gynecologists (n = 456; 66.1 %). In terms of post-graduate education 384 participants had a relative M.Sc. degree (55.6 %), 221 had a Ph.D. degree (31.8 %), 296 had studied abroad (42.9 %) and 295 had performed an accredited subspecialty (42.7 %). Interestingly, less than half of the enrolled subjects worked in public services (n = 292; 42.0 %), although this is the most common healthcare delivery setting in Greece. Baseline demographics are illustrated in Fig. 1.

### 3.2 Perspectives regarding CS rates in Greece

Overall, 12 questions were dedicated to perception regarding the Greek CS rate and what actions should be taken. 29.7 % of the participants believed that the estimated 53.8 % and 58.7 % CS rate in public and private hospitals in Greece, respectively, resulted from adherence to guidelines and was perceived as “reasonable” or “justified” and were deemed personally “acceptable” by 29.7 % of the participants.

**Fig. 2:**
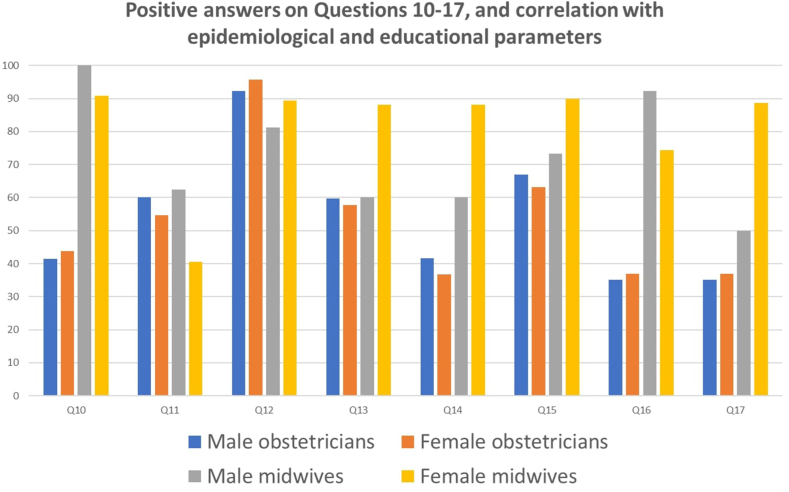
Rates of positive answers among obstetrician's vs midwives and males vs females regarding actions that may improve the CS rates in Greece.

32.0 % of the participants considered the national CS rate as “unavoidable” and that it could not be prevented or stopped. 78.5 % agreed that aggressive measures should be taken to decrease the annual CS rate in Greece (Table 3, Table 4). Sensitivity analysis based on baseline demographics demonstrated that older obstetricians and subjects with a degree obtained abroad were more likely to perceive current CS rates as “reasonable” or “justified” (Table 3) and personally “acceptable” or “unavoidable” (Table 4). Although male midwives were more likely to perceive current CS as “justified”, sex did not influence the opinion that aggressive measures should be definitely taken to decrease the annual CS rate in Greece (Table 4). Additionally, sex did not affect the views on what corrective measures should be undertaken among obstetricians, although female midwives were more likely to advocate for multiple contemporary corrective measures (Table 5, Fig. 2).

**Table 3:** Positive answers on Questions 1–6, comparison of Physicians versus midwives and correlation with epidemiological and educational parameters.

	Q1	Q2	Q3	Q4	Q5	Q6
Questions	Greece rates justified according to obstetrical science guidelines	EU rates justified according to obstetrical science guidelines	USA rates justified according to obstetrical science guidelines	China rates justified according to obstetrical science guidelines	Brazil rates justified according to obstetrical science guidelines	Turkey rates justified according to obstetrical science guidelines
**Physicians vs. Midwives**	31.3 % vs. 31.2 %	**85.6 % vs. 66.7 %**	**78.7 % vs. 50.9 %**	**84.5 % vs. 65.7 %**	22.2 % vs. 24.7 %	31.4 % vs. 29.9 %
***P-value***	0.968	**<0.001**	**<0.001**	**<0.001**	0.5	0.727
**Physician:**						
Age^a^, *P-value*	**0.02**	0.335	0.694	0.311	**0.017**	0.183
Male vs. Female	32.7 % vs. 24.7 %	86.2 % vs. 84.0 %	79.8 % vs. 75.7 %	84.8 % vs. 83.8 %	**24.5 % vs. 12.3 %**	32.0 % vs. 27.4 %
*P-value*	0.14	0.628	0.435	0.832	**0.025**	0.446
**Midwives:**						
Age^a^, *P-value*	0.225	0.787	0.659	0.820	0.753	0.211
Male vs. Female	**6.7 % vs. 33.3 %**	85.7 % vs. 65.0 %	50.0 % vs. 50.6 %	85.7 % vs. 63.9 %	7.1 % vs. 26.3 %	85.7 % vs. 68.5 %
*P-value*	**0.032**	0.114	0.966	0.098	0.11	0.178
**Mcs Yes vs. No**	30.5 % vs. 32.4 %	**82.6 % vs. 73.6 %**	71.9 % vs. 66.8 %	**82.2 % vs. 71.9 %**	22.0 % vs. 23.4 %	31.4 % vs. 29.9 %
***P-value***	0.669	**<0.001**	0.228	**0.004**	0.758	0.703
**Phd Yes vs. No**	30.9 % vs. 33.1 %	**87.4 % vs. 74.0 %**	**80.0 % vs. 64.5 %**	**84.7 % vs.74.2 %**	24.4 % vs. 22.6 %	34.0 % vs. 29.7 %
***P-value***	0.589	**0.001**	**<0.001**	**0.005**	0.654	0.298
**Degree abroad vs. Greece**	**38.8 % vs. 28.6 %**	**87.9 % vs. 76.2 %**	**84.0 % vs. 65.5 %**	**86.7 % vs. 75.8 %**	**30.0 % vs. 20.9 %**	**39.7 % vs 28.6 %**
***P-value***	**0.023**	**0.004**	**<0.001**	**0.011**	**0.036**	**0.028**
**Specialty abroad vs. Greece**	27.0 % vs. 31.9 %	89.7 % vs. 84.2 %	75.4 % vs. 80.2 %	87.4 % vs.83.7 %	20.7 % vs 20.6 %	27.9 % vs 31.4 %
***P-value***	0.312	0.159	0.287	0.434	0.985	0.484
**Subspecialty** **Yes vs. No**	32.8 % vs. 30.0 %	82.1 % vs. 76.3 %	**76.1 % vs. 64.4 %**	80.6 % vs.76.1 %	21.5 % vs. 24.4 %	30.4 % vs 31.7 %
***P-value***	0.448	0.08	**0.002**	0.207	0.418	0.727

a P- values expressing comparisons within 6 age subgroups.

**Table 4:** Positive answers on Questions 7–9, comparison of Physicians versus midwives and correlation with epidemiological and educational parameters.

	Q7	Q8	Q9
Questions	Is current CS rate in Greece acceptable	Is current CS rate in Greece inevitable	More effort is needed to reduce Greece CS rate
**Physicians vs. Midwives**	**27.4 % vs. 19.7 %**	**45.9 % vs. 27.9 %**	87.7 % vs. 86.9 %
***P-value***	**0.04**	**<0.001**	0.76
**Physicians:**			
Age^a^, *P-value*	**0.053**	0.769	**0.006**
Male vs. Female	28.5 % vs. 29.1 %	44.1 % vs. 50.0 %	83.4 % vs. 91.1 %
*P-value*	0.255	0.377	0.072
**Midwives:**			
Age^a^, *P-value*	0.131	0.911	0.083
Male vs. Female	13.3 % vs. 20.3 %	31.3 % vs. 27.5 %	100 % vs. 90.8 %
*P-value*	0.513	0.752	0.218
**Mcs Yes vs. No**	25.2 % vs. 24.6 %	38.1 % vs. 41.9 %	86.9 % vs. 87.7 %
***P-value***	0.878	0.370	0.764
**Phd Yes vs. No**	25.5 % vs. 25.4 %	44.9 % vs. 36.8 %	86.9 % vs. 87.0 %
***P-value***	0.975	0.07	0.955
**Degree abroad vs. Greece**	**31.6 % vs. 22.8 %**	**50.9 % vs. 37.3 %**	82.5 % vs. 88.6 %
***P-value***	**0.05**	**0.008**	0.07
**Specialty abroad vs. Greece**	23.6 % vs. 28.9 %	40.3 % vs. 45.8 %	87.2 % vs. 85.7 %
***P-value***	0.282	0.331	0.681
**Subspecialty** **Yes vs. No**	26.3 % vs. 23.3 %	43.6 % vs. 36.8 %	**84.3 % vs. 89.9 %**
***P-value***	0.413	0.103	**0.04**

a P- values expressing comparisons within 6 age subgroups.

**Table 5:** Positive answers on Questions 10–17, comparison of Physicians versus midwives and correlation with epidemiological and educational parameters.

	Q10	Q11	Q12	Q13	Q14	Q15	Q16	Q17
Questions	Training credits to permit practicing obstetrics	Regular examinations to continue practicing obstetrics	Protocols & guidelines from relevant OB/GYN Colleges	Protocols & guidelines from government with legislation	Keeping guidelines under scrutiny by the government	Personal records of obstetrical practice by authorized Medical Societies	Perform regular VBAC to improve CS rate	Control on first CS indication
**Physicians vs.**	42.1 vs.	**56.1 % vs.**	92.6 % vs.	**59.0 % vs.**	**41.1 % vs.**	**66.1 % vs.**	**36.4 % vs.**	**60.0 % vs.**
**Midwives**	41.5 %	**79.2 %**	88.9 %	**86.2 %**	**84.6 %**	**88.9 %**	**75.8 %**	**85.4 %**
***P-value***	0.89	**<0.001**	0.116	**<0.001**	**<0.001**	**<0.001**	**<0.001**	**<0.001**
**Physicians:**								
Age^a^, *P-value*	0.094	0.052	0.256	0.936	0.655	0.808	0.075	0.483
Male vs. Female	41.5 vs. 43.8 %	60.0 vs. 54.6 %	92.3 vs. 95.8.%	59.7 vs. 57.7.%	41.7 vs. 36,7 %	66.9 vs. 63.2 %	35.1 vs 37.0 %	35.1 vs 37.0 %
*P-value*	0.699	0.349	0.227	0.727	0.384	0.501	0.177	0.790
**Midwives:**								
Age^a^, *P-value*	**<0.001**	0.742	0.099	**0.038**	0.213	0.148	0.657	0.256
Male vs. Female	100.0 vs 90.8	62.5 vs 40.5 %	81.3 vs. 89.4 %	60.0 vs 88.1 %	**60.0 vs 88.1 %**	**73.3 vs 90 %**	**92.3 vs. 74.3 %**	**50.0 vs. 88.7 %**
*P-value*	0.088	**<0.001**	0.316	**0.002**	**0.048**	**0.048**	0.147	0.147
**MSc Yes vs. No**	43.2 vs. 38.1 %	64.7 vs. 63.3 %	92.3 vs. 89.7 %	65.8 vs. 69.7 %	52.3 vs. 60.1 %	73.1 % vs. 75.5 %	46.2 vs. 55.2 %	67.5 vs. 70.2 %
***P-value***	0.19	0.7	0.254	0.287	**0.049**	0.485	0.07	0.461
**Ph.D. Yes vs. No**	41.5 vs. 41.3 %	64.6 vs. 62.0 %	92.6 vs. 90.1 %	59.0 vs. 71.6 %	42.1.6 vs. 61.5 %	68.6 vs. 76.3 %	35.2 vs. 56.1 %	66.7 vs. 69.0 %
***P-value***	0.957	0.518	0.314	**<0.001**	**<0.001**	**0.04**	**<0.001**	0.557
**Degree abroad vs. Greece**	43.0 vs. 41.2 %	51.8 vs. 66.9 %, **<0.001**	91.4 vs. 91.2 %	59.9 vs. 69.6 %	43.0 vs. 58.6 %	62.0 vs. 77.0 %	42.0 vs. 52.4 %	60.3 vs. 70.4 %
***P-value***	0.713	**<0.001**	0.924	**0.028**	**0.001**	**<0.001**	0.092	**0.02**
**Specialty abroad vs. Greece**	55.9 vs. 37.0 %	63.9 vs. 53.6 %	92.6 vs. 92.4 %	58.1 vs. 58.8 %	41.0 vs. 42.3 %	67.7 vs. 67.0 %	44.3 vs. 34.7 %	60.3 vs. 61.7 %
***P-value***	**<0.001**	**0.047**	0.94	0.895	0.801	0.894	0.144	0.778
**Subspecialty** **Yes vs. No**	46.0 vs. 38.0 %	62.5 vs. 64.6 %	92.8 vs. 89.9 %	60.1.vs. 73.4 %	45.5 vs. 63.7 %	68.7 vs. 78.0 %	41.4 vs. 57.6 %	66.4 vs. 69.9 %
***P-value***	**0.04**	0.579	0.201	**<0.001**	**<0.001**	.**007**	**<0.001**	0.346

a P- values expressing comparisons within 6 age subgroups.

### 3.3 Responses regarding actions that should be taken to reduce current national CS rate

Among the various actions proposed to reduce CS rate in Greece, adopting protocols and guidelines from relevant international medical societies (e.g., HOGS) was preferred by 87.6 % of the participants. Keeping procedural records of obstetrical practice by authorized medical organizations, regular examinations to continue practicing, implementation of protocols and guidelines from the government, and rigorous control of the first CS indication were considered appropriate actions by 70.7 %, 61.2 %, 65.4 %, and 65.4 % of the participants, respectively, supported mainly by obstetricians rather than midwives. In contrast, ensuring that the guidelines were under critical observation by the government with federal regulatory mechanisms and performing routinely VBAC was not widely preferred as strategies to reduce CS rates in Greece. Table 5 summarizes the responses regarding actions that should be taken regarding the current national CS rate stratified by several demographic and educational parameters.

### 3.4 Perspectives regarding CS rates in high-, middle- and low-income countries

**Fig. 3:**
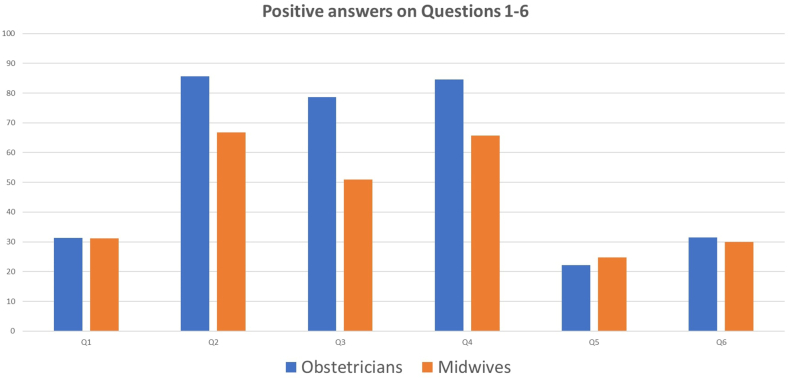
Rates of positive answers among obstetrician's vs midwives regarding justification of CS rates in Q1-Greece (public hospitals 53.8 % and private clinics 58.7 % 2014), Q2- European Union (average 25.3 %), Q3- USA (2007–2014 average 33 %), Q4- China (2007–2014 average 27 %), Q5- Brazil (in 2014 average 57 % and private clinics 80–90 % in2011–2012) and Q6- Turkey (2007–2014 average 37 % and private clinics 66.7 % in 2014).

**Fig. 4:**
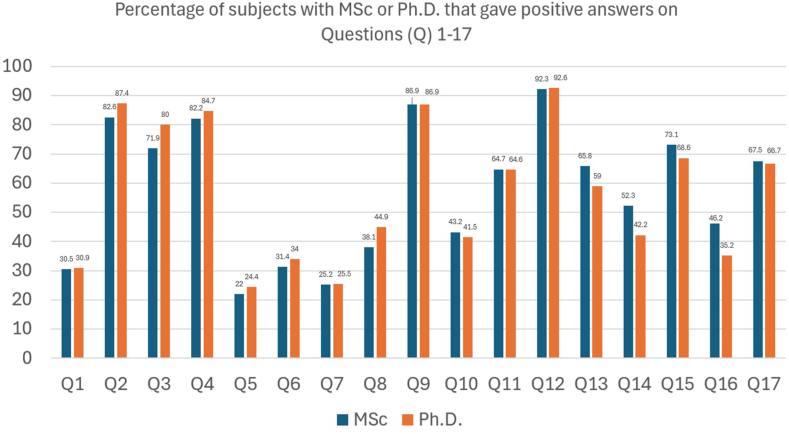
Percentages of subjects with MSc or Ph.D. that gave positive answers on Questions (Q) 1-17.

**Fig. 5:**
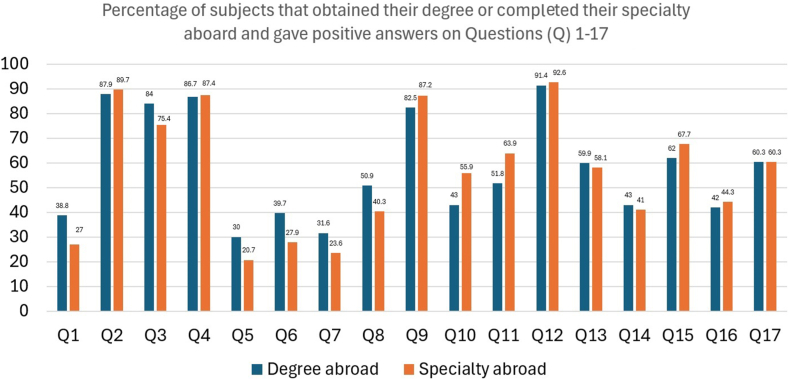
Percentages of subjects that obtained their degree or completed their specialty aboard that gave positive answers on Questions (Q) 1-17.

Overall, 6 questions were dedicated to perceptions regarding the CS rates in high-, middle- and low-income countries. The lower CS rates observed in high-income countries (i.e., Europe 25 %, USA 33 %, China 27 %) were perceived by the majority of the participants as “reasonable” or “justified” and a result of adherence to guidelines (Table 2). The higher CS rates observed in middle- and low-income countries (i.e., Brazil: public hospitals 56 % and private clinics 80–90 %; public hospitals 37 % and private clinics 66.7 %) were perceived as “reasonable” or “justified” only by a minority of the participants (Table 2). Sensitivity analyses demonstrated that obstetricians, compared to midwives and professionals with MCs, PhD, or who had additional education abroad, were more likely to respond positively and justify the reported CS rates (Table 3). The rates of positive answers among obstetrician's vs midwives for the corresponding questions are illustrated in Fig. 3. The percentages of subjects with MSc or Ph.D. and subjects that obtained their degree or completed their specialty aboard that gave positive answers on questions 1–17 are illustrated in Fig. 4, Fig. 5 respectively.

## 4 Discussion

The issue of excessive utilization of surgical and invasive procedures is gaining greater acknowledgment on a global scale. However, country-specific evidence, especially for middle-and low-income countries, is limited. This is the first study to investigate Greek obstetricians' and midwives' perspectives and opinions on CS rates in Greece and worldwide. Most participants perceived the lower CS rates observed in high-income regions as “justified” driven by guideline-directed management. At the same time, they advocated for corrective actions to reduce national CS rates. Nonetheless, about one-third of the participants still considered the high, above WHO standards, Greek CS rate “justified” or “unavoidable”.

Based on a global survey conducted in 2016, the median optimal CS was estimated to be around 20 % (IQR = 15–30 %) of all deliveries [14]. According to the Greek National Organization for the Provision of Health Services, the CS rate 2014 was 53.8 % in public hospitals and 58.7 % in private clinics. In 2016, WHO representatives met with members of the Greek Ministry of Health to reduce the rate of CS in Greece, as it was estimated that over half of the births in the country were due to CS [8]. Nevertheless, in 2017, Greece was one of the few countries that failed to provide any national data to Eurostat, highlighting the absence of reporting data as one of the fundamental reasons for the high CS rate in Greece [5,13]. However, our results show that most Greek obstetricians/gynecologists and midwives are aware of the existing problem of the unacceptably high CS rate and agree that there is an imperative need for change.

A combination of economic, organizational, social, cultural, and even medico-legal factors, rather than strictly evidence-based medicine, seems to drive the high CS rates globally [15]. The most important ones that have been reported include safety for both the fetus and mother, disbelief in vaginal delivery skills [15], financial incentives, especially in private institutions [16,17], fear of litigation from malpractice [18], a desire to enhance patient satisfaction [[18], [19], [20]], personal experience and preference [21], convenience of scheduling, and staffing patterns in private facilities [17,22]. However, experts can argue that, specifically for Greece, the high CS rate can be potentially justified by the higher-than-expected perinatal mortality rates that have been documented for the last 20 years, which can be partially attributed to the early recourse to CS, especially considering the ongoing economic crisis of the country [23].

To identify realistic strategies to improve high CS rates, the viewpoints of the respondents regarding potential solutions were analyzed. The most favored of these solutions were the adoption of protocols and guidelines from relevant colleges, medical societies, or the government; keeping personal records of obstetrical practice by authorized medical organizations; regular examinations to continue their practice; and rigorous control of the first CS indication. In contrast, keeping guidelines under the scrutiny of the government and performing vaginal delivery after cesarean section (VBAC) were not widely preferred solutions.

Governments and experts in this field have proposed similar solutions. Specifically, the obligation of hospitals and counties to publicly report their CS rates and investigate their performance against the guidelines, financial incentives to reduce CS rates, economic support of low-income countries to raise the CS rate above 10 %, and the use of a uniform classification system and clinical guidelines for CS have all been proposed [4,23,24]. The nationwide application of the Robson classification could assist in identifying the significant contributors to high CS rates [25,26], mainly targeting private facilities and women with a history of previous C-sections [22]. Furthermore, the increase in the number of midwifery staff to provide one-to-one care in labor and the provision of unbiased information to women regarding the benefits and risks of CS [18], as well as the improvement of skills related to vaginal obstetric procedures and pain-free vaginal delivery, seemed to be additional reasonable solutions [27].

Although these interventions have already been implemented, their suboptimal effect has been suggested due to the complexity of the factors that drive the underuse and overuse of CS worldwide, as well as the prevalent approach in research to focus on single interventions that target only one aspect [28]. A confirmation of the above, which is also in concordance with the opinion of our cohort that stricter regulations by the government as a solitary measurement will not be effective toward CS rate control, is the example of Turkey (middle-, low-income country), where the CS rate increased from 47.5 % in 2011 to 54.4 % in 2019, despite the government initiative to pass a new law to limit births by CS [4].

A significantly lower percentage of midwives considered CS rates in Greece acceptable and favored lower CS rates, which was in line with similar studies conducted in Sweden, Australia, and Italy [18,20,29]. Overall, midwives proposed significantly more interventions to successfully reduce the CS rate. Additionally, midwives were more likely to offer VBAC vs CS section, a finding consistent in other European countries [30,31]. There was no significant difference between male and female physicians in their attitudes toward CS, which is in contrast with the observations from China, where most female obstetricians and midwives reported CS as the preferred mode of delivery, half of whom had no medical indications [32], and the observations from Sweden, where male obstetricians accepted a higher level of CS than their female colleagues [31]. This might have been affected by the fact that male and female representation was almost equal in the Greek cohort.

### 4.1 Limitations

The results of the present survey should be interpreted in the context of several limitations. First, although the Greek CS rate used as a reference in the questionnaire was based on data from the Greek National Organization for the Provision of Health Services, Greece is one of the few countries that has not officially reported any data regarding CS rates to Eurostat or other official databases [13]**.** Considering there is no obligation for private and public hospitals in Greece to report their CS rates, the reference CS rates in Greece in this survey might be inaccurate and even higher than those noted. Second, the response rate of our online survey could not be calculated because the number of obstetricians and midwives who received the invitation via email is unknown. Third, the data used for this analysis were gathered over a relatively long period of time, starting a couple of years ago, nonetheless the results are unlikely to have undergone significant changes over this period. Additionally, selection bias might have affected the study findings, as the participants might not have been representative of all obstetricians/gynecologists and midwives performing CS in Greece, precluding the possibility of generalizing our results. Finally, the preferred mode of delivery (CS versus vaginal delivery) in the participants’ daily practice was not investigated, although 15 % reported that they were not actively involved in obstetrics, which could affect their perceptions toward CS.

## 5 Conclusion and future directions

This study showed that, although both Greek obstetricians/gynecologists and midwives were aware of the unacceptably high CS rates, midwives considered the current CS rates unjustified to a greater degree. The fact that Greek CS rates (>50 %) were considered "acceptable" by 29.7 of the participants, highlights that this should be considered as an "acceptable" strategy towards the "wrong direction". Additionally, despite the lack of national data, most participants agreed that there is room for improvement, with midwives recommending significantly more concurrent interventions to reduce the current national CS rate successfully. Overall, the need for standardized re-certification examinations and the creation of qualitative initiatives to control guideline adherence in both public and private health care settings was underlined by both obstetricians and midwives. Additionally, research with potential head-to-head comparisons of data from high-vs. middle-/low-income countries is needed to identify differences in healthcare systems and the respective control mechanisms to improve clinical practice and utilization of resources globally. Further investigation is warranted to explore the impact of educational interventions and training programs targeting pregnant women, physicians, and midwives.

## CRediT authorship contribution statement

**Lioumpov Tonakanian:** Writing – review & editing, Writing – original draft, Visualization, Validation, Software, Resources, Project administration, Methodology, Investigation, Funding acquisition, Formal analysis, Data curation, Conceptualization. **Stamatios Petousis:** Writing – review & editing, Formal analysis, Data curation. **Panagiotis Volteas:** Writing – review & editing, Writing – original draft, Formal analysis, Data curation. **Aikaterini Karavida:** Writing – review & editing. **Konstantinos Dinas:** Writing – review & editing. **Theodoros Theodoridis:** Writing – review & editing. **Alexandros Sotiriadis:** Writing – review & editing. **Apostolos Athanasiadis:** Writing – review & editing, Resources, Project administration, Methodology, Funding acquisition, Formal analysis, Conceptualization.

## Data availability statement

The data that support the findings of this study are available from the corresponding author upon reasonable request.

## Fundingstatement

This research received no specific grant from any funding agency in the public, commercial, or not-for-profit sectors.

## Declaration of competing interest

The authors declare that they have no known competing financial interests or personal relationships that could have appeared to influence the work reported in this paper.

## Abbreviations

CS: Cesarean Section
VBAC: Vaginal Delivery After Cesarean Section
Ph.D: Doctor of Philosophy
